# Insights into the Pollutant Removal Performance of Stormwater Green Infrastructures: A Case Study of Detention Basins and Retention Ponds

**DOI:** 10.3390/ijerph181910104

**Published:** 2021-09-26

**Authors:** Seol Jeon, Siyeon Kim, Moonyoung Lee, Heejin An, Kichul Jung, Myoung-Jin Um, Kyungjin An, Daeryong Park

**Affiliations:** 1 Department of Civil, Environmental and Plant Engineering, Konkuk University, Seoul 05029, Korea; louie317@konkuk.ac.kr (S.J.); kimsiyeon@konkuk.ac.kr (S.K.); moon0e@konkuk.ac.kr (M.L.); gmlwls98@konkuk.ac.kr (H.A.); 2 Division for Integrated Water Management, Korea Environment Institute, Sejong 30147, Korea; kcjung@kei.re.kr; 3 School of Smart City Engineering, Kyonggi University, Suwon 16227, Korea; mum@kgu.ac.kr; 4 Department of Forestry and Landscape Architecture, Konkuk University, Seoul 05029, Korea; 5 Department of Civil and Environmental Engineering, Konkuk University, Seoul 05029, Korea

**Keywords:** green infrastructure (GI), total suspended solids (TSS), total phosphorous (TP), ordinary least squares (OLS), multilinear regression (MLR)

## Abstract

The quality of water has deteriorated due to urbanization and the occurrence of urban stormwater runoff. To solve this problem, this study investigated the pollutant reduction effects from the geometric and hydrological factors of green infrastructures (GIs) to more accurately design GI models, and evaluated the factors that are required for such a design. Among several GIs, detention basins and retention ponds were evaluated. This study chose the inflow, outflow, total suspended solids (TSS), total phosphorus (TP), watershed area, GI area (bottom area in detention basins and permanent pool surface area in retention ponds), and GI volume (in both detention basins and retention ponds) for analysis and applied both ordinary least squares (OLS) regression and multiple linear regression (MLR). The geometric factors do not vary within each GI, but there may be a bias due to the number of stormwater events. To solve this problem, three methods that involved randomly extracting data with a certain range and excluding outliers were applied to the models. The accuracies of these OLS and MLR models were analyzed through the percentage bias (PBIAS), Nash-Sutcliffe efficiency (NSE), and RMSE-observations standard deviation ratio (RSR). The results of this study suggest that models which consider the influent concentration combined with the hydrological and GI geometric parameters have better correlations than models that consider only a single parameter.

## 1. Introduction

The water quality of stormwater runoff has rapidly deteriorated due to urbanization. Consequently, stormwater runoff introduces significant quantities of nonpoint-source pollutants into water bodies in the regions surrounding urban areas. As a result, different types of stormwater best management practices (BMPs), which are referred to as green infrastructure (GI) and low-impact development (LID), have been developed in order to address stormwater runoff and its reduction in quality. For instance, Yu et al. [1] presented BMP designs for parameters, such as antecedent dry days and hydraulic conditions (hydraulic loading rate and hydraulic retention time), as well as existing factors, such as the BMP geometry and hydrological factors.

Constructed stormwater wetlands (CSWs) are a common form of stormwater BMP. However, the plug flow assumptions in first order (the k-C* model) decay kinetics do not accurately reflect the real fluid dynamics in CSWs [2]. Real fluid dynamics combine plug flow and mixed flow. It is difficult to estimate the accurate combination of plug and mixed flows. This provides uncertainty in effluent estimation. Nevertheless, most previous studies continued to use plug flow-based k-C* models in conjunction with univariate analysis. In the case of the k-C* model, it was possible to estimate the treatment performance conservatively when the background concentration was considered. These models employ parameters, such as the residence time or flow velocity, in which there is no single unique value, and, in some cases, the distribution of the wetland residence time alone deviates from the plug flow behavior. Therefore, although the k-C* model is simple and widely used, it does not sufficiently characterize the complex processes occurring in wetlands and CSWs that result in the reduction of nonpoint-source pollutants [3].

Barrett [4] investigated the performance of retention ponds using the BMP database and showed that the effluent concentration (C_out_) of retention ponds tended to decrease as the permanent pool volume increased, but that the volume divided by the mean annual storm runoff volume at the site was not significant for fewer than 10. However, even with multiple linear regression (MLR), the relationship between the pool volume and water quality reduction could not be accurately expressed. A report by the Environmental Protection Agency (EPA) [5] indicated that the removal rate of total suspended solids (TSS) increased as the surface area contributing to the watershed increased, but after some increase, the effect of the surface area on the TSS reduction diminished. According to Barrett [4], however, in contrast to the EPA report, TSS removal was not significantly related to the surface area. Barrett [6] evaluated both basic design characteristics and water quality data in the BMP database and stated that pollutant concentrations are determined and related to the site design. The percent reduction in the pollutant concentration observed throughout the system is commonly used to calculate the BMP pollutant removal efficiency. Nevertheless, Strecker et al. [7] recognized that this approach is unreliable since the percent reduction is highly dependent on the influent concentration (C_in_) and other parameters, including the GI geometric and hydrological factors. In the case of retention ponds, the greater the permanent pool volume is, the smaller the variability in the discharge concentrations and the better the performance for individual events with a large runoff volume. A significant relationship was not observed in the data among the detention time, depth of banks and TSS removal.

According to previous studies, the stormwater treatment performance in wetlands, a type of storage GIs, is determined primarily by measuring the inflow or hydraulic loading rate (HLR) and the detention time, both of which are influenced by the storm intensity, runoff volume, and surface area and volume of GIs [8,9,10,11]. Carleton et al. [12] investigated the wetland factors’ influence on stormwater treatment performance in 35 studies considering 46 wetland systems. In particular, Strecker et al. [13] suggested that the area ratio may not be more important than the ratio of the average runoff volume to the storage volume when determining the treatment performance [12]. In addition, Shamma et al. [14] found that the detention time, detention volume, and geometric considerations influence the treatment performance when designing detention facilities; in the case of detention basins, particles remove suspended solids during the detention time by gravity. The settling velocity is related to the GI geometric parameter depth and detention time. Papa et al. [15] found that as the pond depth decreases, the time required for particle sedimentation decreases, which helps to improve the pollutant removal performance. Park et al. [16] found that the effect of retention ponds on the TSS removal efficiency did not differ from that of detention basins; however, in both of these GIs, the TSS effluent concentration decreased as the surface area increased, thus affecting the retention ponds’ performance. Moreover, in the case of detention basins, the influent concentration had a more noticeable effect on the effluent concentration than did the surface area and inflow, whereas in retention ponds, the GI surface area and inflow affected the effluent concentration. Gilliom et al. [17] developed and evaluated a linear model that predicts the effluent concentrations for BMPs and stormwater pollutants using the influent concentrations in the water quality data from the BMP database. They applied four linear models (percent reduction, ordinary least squares (OLS) regression, a robust Theil–Sen estimator, and a linear decay model with an irreducible concentration parameter) and modeled the percent bias (PBIAS), Nash-Sutcliffe efficiency (NSE) and the root mean square error-observations standard deviation ratio (RSR). For both BMPs and stormwater pollutants, none of the four models performed well, although the OLS regression model showed the best overall performance for contaminants, namely, total phosphorus (TP) and TSS, with acceptable PBIAS and good RSR values.

These studies analyzed GI performance based on influent concentration alone, and did not consider design characteristics, such as GI geometric and hydrological factors themselves. Most previous models for designing GIs were developed considering only the influent and effluent concentrations. However, these models encounter difficulties when the concentration range is outside the design configuration, such as extrapolation beyond the contaminant concentrations that have been previously measured. In reality, many factors affect the GI performance, but which parameters exert a greater influence remains unclear. Additionally, no quantitative evaluations are available, and previous studies predicted only the stormwater runoff effluent concentration as the influent concentration, while excluding other parameters.

The aim of this study is to assess how GI design factors, such as GI geometric and hydrological factors, influence performance, compared to influent concentration. Hence, the purpose of this study is to investigate how geometric, hydrological, and influent concentration factors of GIs influence the effluent concentration. Through model evaluations of detention basins and retention ponds, this study uncovers which parameter combination exhibits the best performance for each GI, and a better model is found by comparing the developed model with a model that only considers the influent concentration. The results of this study are expected to provide insight into the influencing factors in the design of GIs.

## 2. Methods

### 2.1. Stormwater BMP Database

**Figure 1:**
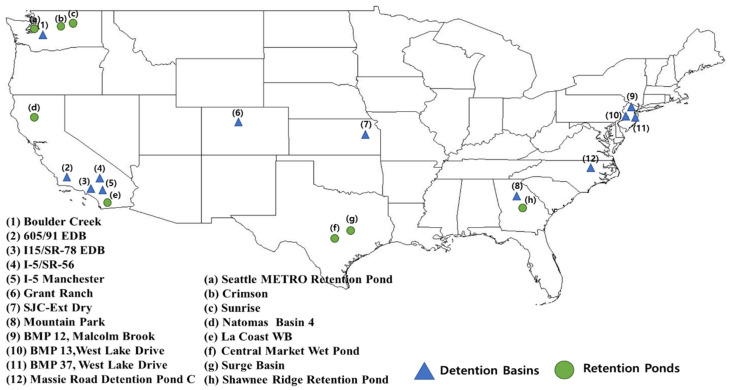
Locations of the detention basins and retention ponds used in this study.

The detention basin and retention pond data were acquired from the BMP database, and the influent flow rates and geometric factors of the two GIs were sorted. Essentially, only the stormwater events listed that include the GI geometry (area, depth, and volume), hydrological factors (discharge, detention time, and hydraulic loading rate), watershed area, inflow concentration and outflow concentration were used. Ultimately, data from a total of twelve detention basins and eight retention ponds were used (Figure 1). Because the number of stormwater events differs for each GI, bias can occur. In addition, for each stormwater event, the GI geometry remains unchanged, while the remaining parameters vary, resulting in increased variability of the model. If all of these data are used in the model construction, good performance would not be achieved, so the model performance is validated with three sampling methods.

Not all stormwater events collected from the BMP database were used; only those occurring when the GI was operating properly were chosen. To satisfy this criterion, the inflow rate must be greater than the outflow rate, and the inflow concentration must be greater than the outflow concentration [16]. All data were subjected to normalization and standardization because the units and value sizes differed among the various parameters and, thus, could not be compared.

The aim of this study is to assess if the influent concentration is dominant in the design of detention basins and retention ponds as GIs. The reason for choosing traditional techniques (detention basins/retention ponds) among GIs is that the quantity of stormwater monitoring data about these techniques is the largest in the BMP database. In the stormwater BMP/LID database, the number of observed TP is enough to apply statistical analysis, but enough orthophosphate (SRP) data is not available. Therefore, this study applied TP rather than SRP.

**Figure 2:**
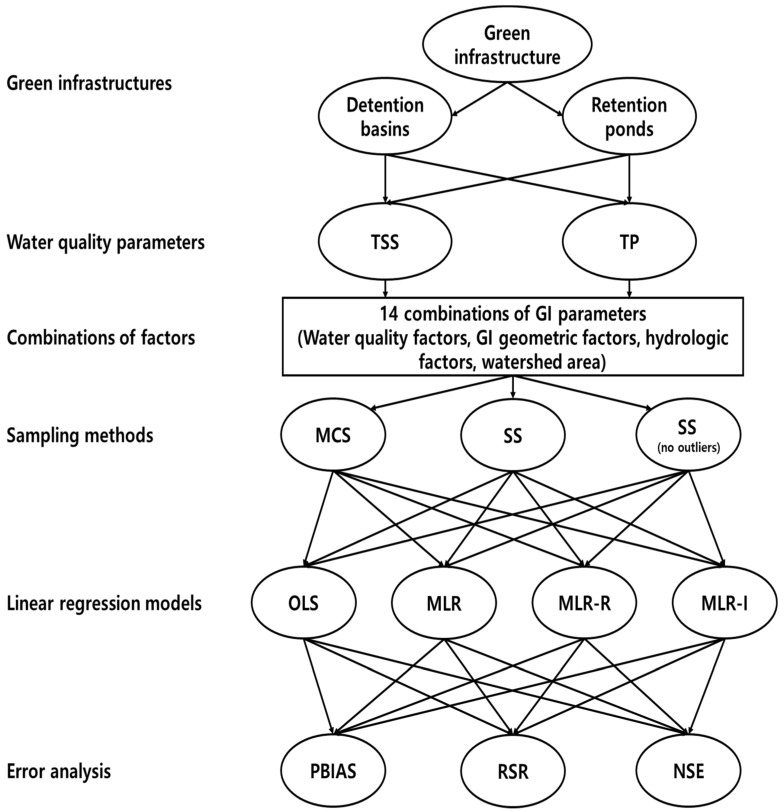
Schematic diagram of the methodology employed in this study.

Sampling without replacement was performed a thousand times for each combination of parameters used in the model. Each combination of parameters required the inflow concentration. In addition, when only one element was added, one of the following was included: the hydraulic loading rate (HLR), inflow rate (Q), detention time (T), area (A), or ratio of the area to the watershed area (A/WA). When two additional factors were added, the GI geometry, watershed area (WA), or A/WA was included, along with a hydrological factor, such as the inflow rate (Q) or detention time (T). Excluding the OLS regression model, which considers only the inflow concentration, a total of 14 combinations were established to evaluate the model. Figure 2 depicts a flow chart of the methodology used in this study.

### 2.2. Sampling Methods

#### 2.2.1. Modified Cluster Sampling (MCS)

Cluster sampling is a method in which all elements are sampled by selecting a specific cluster after creating clusters according to certain criteria. Each cluster encompasses a naturally occurring group. However, this approach is less accurate than simple random sampling because the elements which are not organized into clusters during the cluster selection phase are not included in the sampling scheme [18]. To compensate for this flaw, random sampling is carried out in all clusters; this approach is similar to stratified sampling but excludes the selection of clusters. This step is expected to compensate for the lack of accuracy.

**Figure 3:**
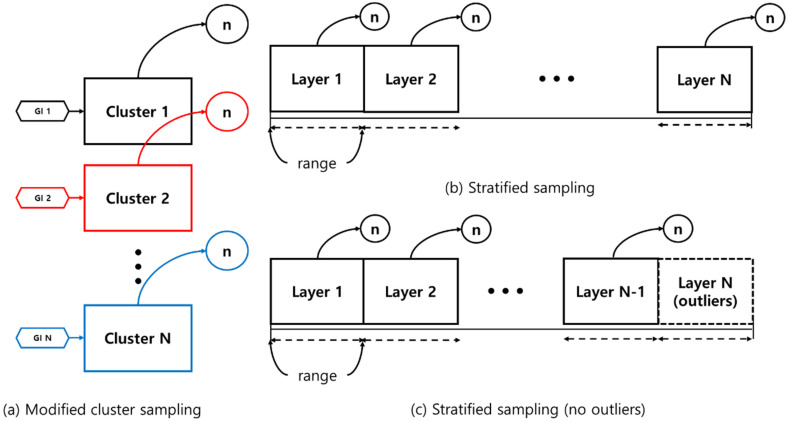
Schematic diagram of the three sampling methods. (**a**) Modified cluster sampling (**b**) Stratified sampling (**c**) Stratified sampling (no outliers).

The MCS method involved dividing the population into clusters and then randomly extracting the number of similar stormwater events that were possible for each cluster. The detention basins were extracted by setting the number of events as close as possible for each of the 12 GIs; the eight retention ponds were extracted in the same way. This sampling method is possible only when the GI geometry and the watershed area are separate. In contrast, the hydrological factors and influent concentrations were not separate because they are not constants in a given GI. This approach is suitable for stratified sampling or modified stratified sampling excluding outliers (Figure 3a).

#### 2.2.2. Stratified Sampling

Stratified sampling produces a representative sample when the sample to be collected from the data is not homogeneous, requiring a layered sampling approach. This method samples each layer by splitting the layers into subcollections that have more uniform populations, and more reliable estimates for each sample are obtained if the data are divided by stratification. This sampling technique obtains an estimate of the population in each stratum when each variable is more accurate. Layered sampling gives relatively accurate and informative data on the sample (Figure 3b). The researcher’s knowledge and expert judgment are used to determine the strata, thereby providing objective information about the structure, and the stratification scheme is determined by the estimated population characteristics [18].

As shown in Table 1, stratified sampling was performed by selecting a unit in which the number of stormwater events per parameter was as uniform as possible. The layers created through units can be sampled by creating layers based on hydrological factors and influent concentrations, as well as the GI geometry and watershed area.

#### 2.2.3. Stratified Sampling (No Outliers)

The EPA [5] report indicates that if the area of a retention pond exceeds a certain level, the TSS removal rate does not have a significant effect. In the stratified sampling of all the parameters, which were chosen based on the EPA report, the above stratified sampling method was performed after excluding the layer with the highest unit value at the time of stratified sampling (Figure 3c).

### 2.3. Linear Model Regression

#### 2.3.1. OLS Regression

OLS regression is a type of generalized linear modeling that can be used to model a single response variable on at least an interval scale [19]. The least squares method indicates when the square of the prediction error is minimal, as shown in Equation (2) below:

(1) $$\hat{y}\;={\;\beta }_{0}+\beta_{1}x_{1}+\epsilon$$

(2) $$\sum^{\;}{\left(y-\hat{y}\right)}^{2}\;=\;\mathrm{Least}$$

where y is the dependent variable and x is the independent variable. $\hat{y}$ is the value of y predicted by the model, and ϵ is the prediction error. The regression coefficients are $\beta_{0}$ and $\beta_{1}$. In this study, y is the outflow concentration and x is the inflow concentration in a given stormwater event in the BMP database.

#### 2.3.2. MLR

MLR identifies the effects of the interaction between certain variables independent of the variation in the dependent variable in order to determine the independent variables that best predict or explain the most variance in the dependent variable. In MLR, a dependent variable is related to two or more independent variables, as follows:

(3) $$\hat{y}\;={\;\beta }_{0}+\beta_{1}x_{1}+\beta_{2}x_{2}+\cdots +\beta_{n}x_{n}+\epsilon$$

where y is the dependent variable, $x_{n}$ denotes an independent variable, ϵ is the prediction error, and $\beta_{n}$ denotes the regression coefficients. The inflow concentration must be an independent variable, and additional parameters (including the GI geometric and hydrological factors) are included. When three or more independent variables are entered, one variable from each category is entered because both the GI geometric factors and the hydrology factors are required during the design of GIs.

#### 2.3.3. MLR-Interactions

The magnitude of the relationship between one predictor and the criterion varies as a function of at least one other predictor in an interaction. Generally, one predictor is considered to be a focal predictor, while all other predictors multiplied by the focal predictor are moderators, which are assumed to influence the relationship between the focal predictor and the criterion (although this distinction is arbitrary given the symmetry of the interaction) [20]. This interaction effect is represented as a regression equation via the product of two or more independent variables, as follows:

(4) $$\hat{y}\;=\;{\;\beta }_{0}+\beta_{1}x_{1}+\beta_{2}x_{2}+\beta_{n}x_{n}+\cdots +\beta_{12}x_{1}x_{2}+\cdots +\beta_{\left(n-1\right)n}x_{n-1}x_{n}$$

where ŷ is the predicted value of a dependent variable, $x_{n}$ denotes the independent variables, and $\beta_{n}$ denotes the regression coefficients. A regression coefficient corresponding to the interaction effect of $x_{n-1}$ and $x_{n}$ is $\beta_{\left(n-1\right)n}$, and $x_{n-1}x_{n}$ is the interaction.

#### 2.3.4. MLR-Robust Fit

When outliers exist, linear regression analysis using the least squares method is not suitable. In a robust regression model, outliers are not excluded, and a robust analysis is performed and is not affected if outlier data exist. Robust regression, as the name implies, is a more robust version of least squares regression compared to other regression techniques. When outliers exist, they provide regression coefficient estimates. However, in the absence of outliers, MLR coupled with OLS exhibits better performance than robust regression.

The robust regression model assigns a weight to each data point. The bisquare weight function uses the least squares method to iteratively reweight the data. Robust fitting was conducted with a default tuning constant of 4.685 and the bisquare weight function, which is also called the biweight function, as follows:

(5) $${\mathrm{bisquare}}^{\prime }:w\;=\;\left(\left|r\right|<1\right)\times {\left(1-r^{2}\right)}^{2}$$

(6) $$r\;=\;\frac{\mathrm{resid}}{\mathrm{tune}\times s\times \sqrt{1-h}}$$

where resid is a vector of residuals from the previous iteration, tune is an adjustment constant divided by the residual vector before calculating the weight, h is a vector of leverage values from least squares fitting, and s approximates the standard deviation of the error term given by s = MAD/0.6745, where the MAD of the residuals is their median absolute deviation from the median. For a normal distribution, a constant of 0.6745 makes the estimate unbiased [21,22,23,24].

In this study, MATLAB software was used to analyze the data. Four regression models (OLS, MLR, MLR-Interactions, and MLR-Robust) were created, and model evaluations were performed using the ‘fitlm’ function in the Regression Learner Toolbox.

### 2.4. Model Evaluations (PBIAS, RSR, NSE)

#### 2.4.1. Root Mean Square Error-Observations Standard Deviation Ratio (RSR)

This study normalized the data to evaluate the performance of the four models. The RSR is a ratio based on the observed standard deviation of the root mean square error (RMSE), a commonly used error index statistic. The RSR varies from an optimal value of zero (indicating that the residual variation is zero) to positive infinity. A lower RSR value (close to zero) indicates good model performance with a low RMSE [25,26,27,28]. The RSR is computed as follows:

(7) $$\mathrm{RSR}\;=\;\frac{\mathrm{RMSE}}{{\mathrm{STDEV}}_{\mathrm{obs}}}\;=\;\frac{\sqrt{\sum {\left(Y_{\mathrm{obs}}-Y_{\mathrm{sim}}\right)}^{2}}}{\sqrt{\sum {\left(Y_{\mathrm{obs}}-Y_{\mathrm{mean}}\right)}^{2}}}$$

where $Y_{\mathrm{obs}}$ denotes the observation data, $Y_{\mathrm{sim}}$ denotes the model simulation data, and $Y_{\mathrm{mean}}$ is the mean of the observation data.

#### 2.4.2. Percent Bias (PBIAS)

The PBIAS represents the average trend in which the value of the simulated data is greater or less than the value of the observed data, thereby reflecting the relative error from the measured value. The optimal PBIAS value is zero, and the closer the value is to zero, the more accurate the model simulation is. Positive values indicate that the model exhibits an underestimation bias, and negative values conversely indicate an overestimation bias [28,29]. The PBIAS is computed as follows:

(8) $$\mathrm{PBIAS}\;=\;\frac{\sum \left(Y_{\mathrm{obs}}-Y_{\mathrm{sim}}\right)}{\sum Y_{\mathrm{obs}}}$$

#### 2.4.3. Nash-Sutcliffe Efficiency (NSE)

The NSE normalizes the relative magnitude of the residual variance between the observed value and the model-simulated value, thereby indicating how well a plot of the two data values fits along the 1:1 line [30]. The optimal NSE value is one, and acceptable NSE values are generally between zero and one [28].

(9) $$\mathrm{NSE}\;=\;1-\frac{\sum {\left(Y_{\mathrm{obs}}-Y_{\mathrm{sim}}\right)}^{2}}{\sum {\left(Y_{\mathrm{obs}}-Y_{\mathrm{mean}}\right)}^{2}}$$

Table 2, which is based on figures provided in the work of Gilliom et al. (2020) [17], shows the criteria thresholds for good, acceptable, and poor model performance.

## 3. Results

When designing the GI model, 14 combinations of parameters were created using the GI geometric and hydrological factors, as well as the inflow concentration. The GI geometric factors included the area (A), volume (V), and depth (D), while the hydrological factors included the inflow rate (Q), hydraulic loading rate (HLR), and detention time (T). The watershed area (WA) and the ratio of the GI area to the watershed area (A/WA) were also entered as combination parameters. Among the GI geometric factors, ‘A’ describes the bottom area of a detention basin and the permanent surface area of the pool in a retention pond. Table 3 matches the parameters with the 14 combinations and their numbers.

### 3.1. GI Performance

**Figure 4:**
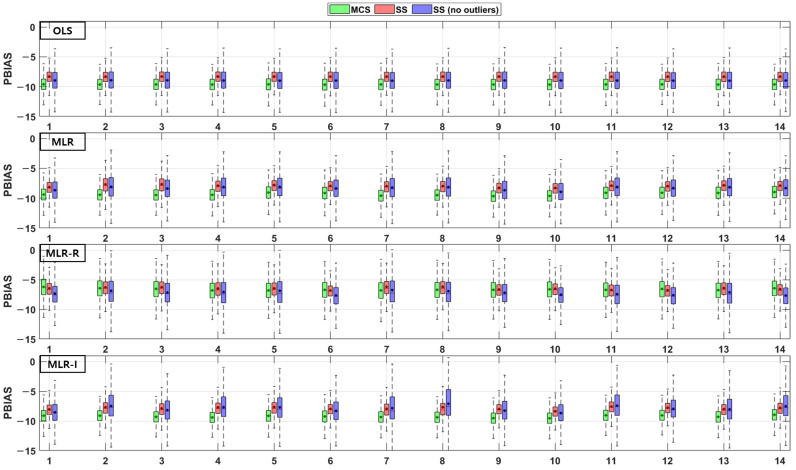
Error analysis (PBIAS) results of TSS removal in detention basins.

**Figure 5:**
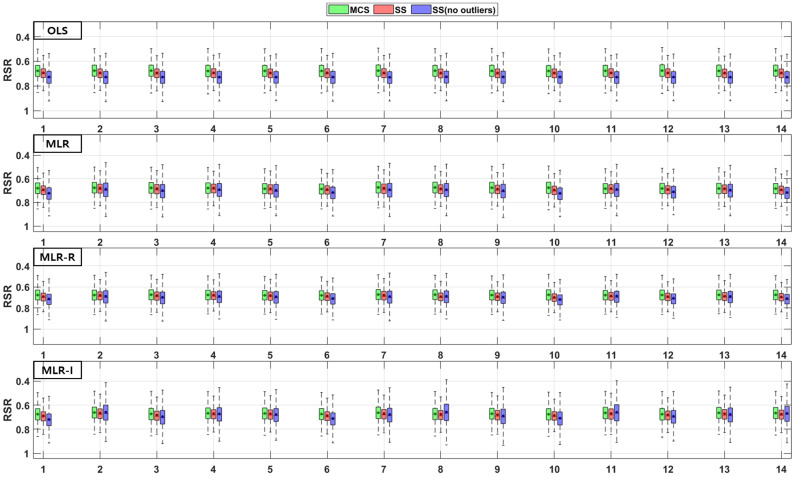
Error analysis (RSR) results of TSS removal in detention basins.

**Figure 6:**
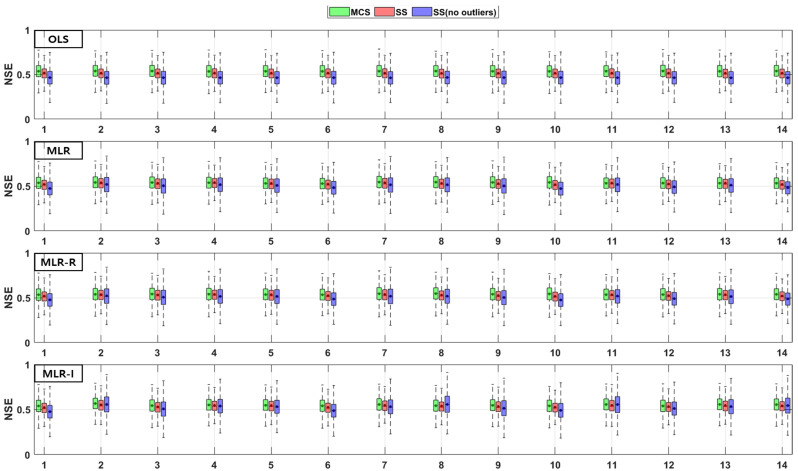
Error analysis (NSE) results of TSS removal in detention basins.

The TSS and TP removal performances in the model evaluations of the detention basins are both “good” and “acceptable”. The removal performance of the detention basins is well represented by each of the MLR models for most parameter combinations. The numbers along the x axes in the figures denote the combination numbers of the parameters listed in Table 3. The TSS removal PBIAS in detention basins ranges from −15% (“acceptable”) to 0% (“good”) (Figure 4). Only the MLR-R model with the MCS method is different from the other models. With the MCS method, the MLR-R model PBIAS is closer to zero than the PBIAS of the other sampling methods. With the SS and SS (no outliers) methods, the MLR-I model PBIAS is close to zero, and the TSS removal performances in the detention basins are “good” (RSR < 0.8) and “acceptable” (RSR < 0.98). The parameter combination results of the sampling methods in the MLR-R model are similar to those in the OLS model (Figure 5). The NSE of TSS removal in the detention basins is mostly “acceptable”, and some parameter combinations in the MLR models exhibit “good” performance. With the SS (no outliers) method, gaps in the resulting values from the MLR-I and OLS models are observed that are not found in the other models. In particular, the NSE with the SS (no outliers) method in the MLR-I model is slightly closer to one (Figure 6).

**Figure 7:**
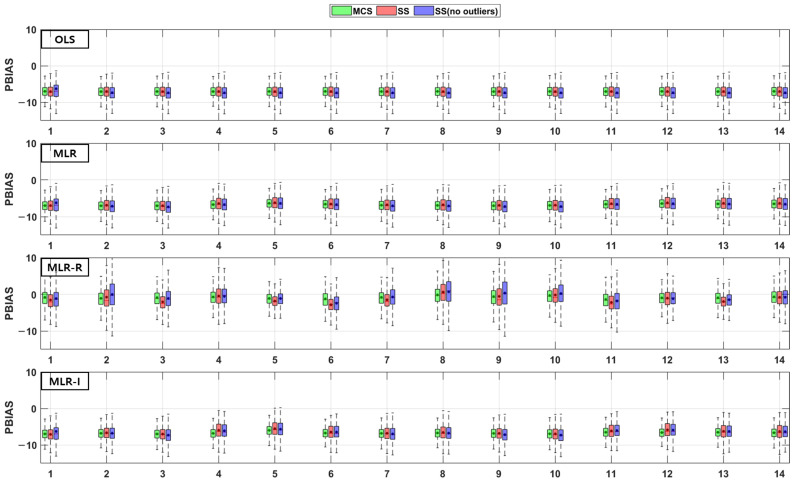
Error analysis (PBIAS) results of TP removal in detention basins.

**Figure 8:**
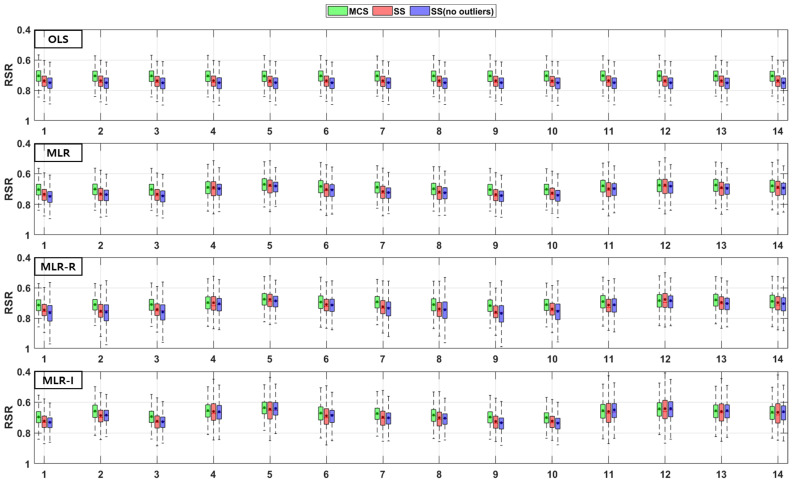
Error analysis (RSR) results of TP removal in detention basins.

**Figure 9:**
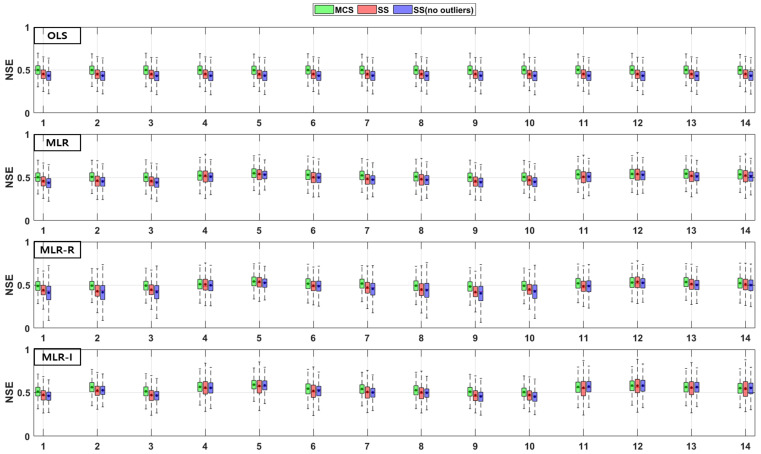
Error analysis (NSE) results of TP removal in detention basins.

The PBIAS TP removal performance values of all the models (except the MLR-R model) in the detention basins are similar to the PBIAS TSS removal performance values in the detention basins. The MLR-R model PBIAS is the closest to zero, indicating a “good” performance. Some of the simulated values of the MLR-R model are overestimated, which is different to the other models (Figure 7). In contrast, the MLR-R model RSR is found to be worse at removing TP for some of the parameter combinations in the detention basins than the OLS model. The MLR-I model RSR, as shown in Figure 8, is closer to zero than the OLS model RSR. Moreover, in terms of the NSE, the TP removal performance in the detention basins is mainly at “good” and “acceptable” levels. The performance reaches a “poor” level for some combinations with the SS (no outliers) method in the MLR-R model (Figure 9).

**Figure 10:**
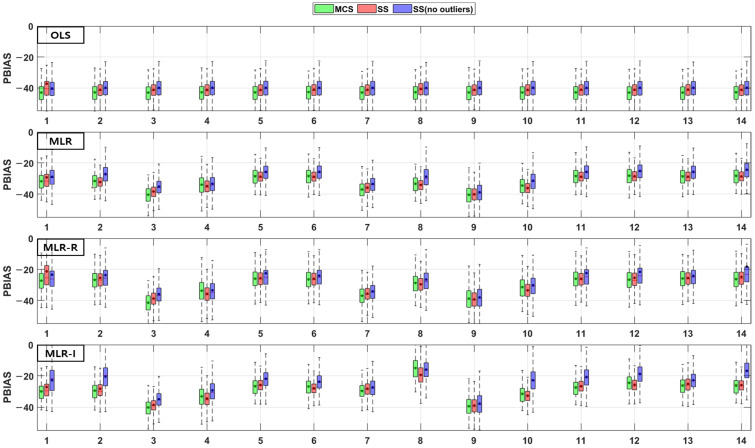
Error analysis (PBIAS) results of TSS removal in retention ponds.

**Figure 11:**
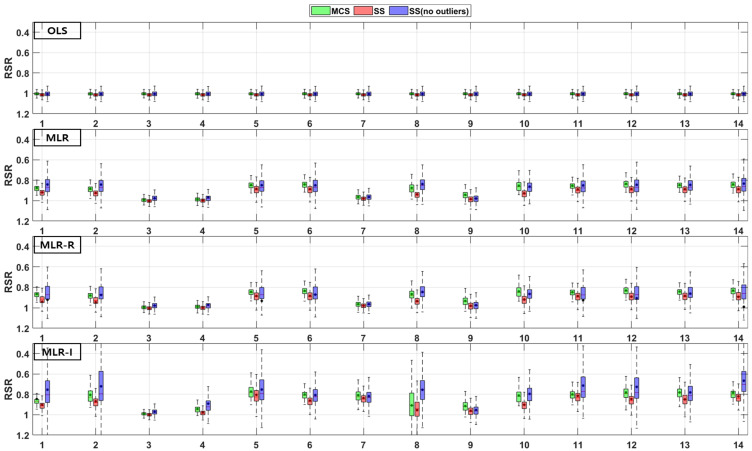
Error analysis (RSR) results of TSS removal in retention ponds.

**Figure 12:**
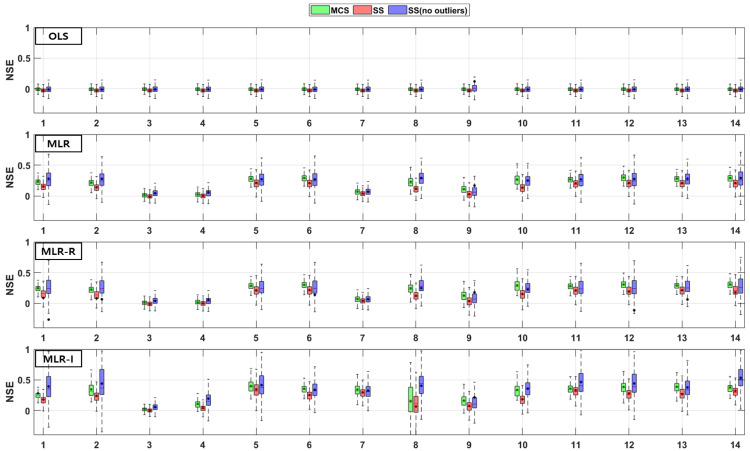
Error analysis (NSE) results of TSS removal in retention ponds.

The TSS and TP removal performances in the retention pond model evaluations are “acceptable” and “poor”, but some of the parameter combinations achieve “good” levels. The TSS removal performance in the retention ponds has a larger variation than that in the detention basins. The PBIAS levels of TSS removal in the retention ponds are “acceptable” and “poor” since the results are lower than −20% (Figure 10). The RSR results of the OLS model for all three sampling methods in Figure 11 are close to one. In addition, the results of the three MLR models for four parameter combinations are similar to those of the OLS model, but the ranges for the other parameter combinations are closer to zero than those of the OLS model (Figure 11). As shown in Figure 12, the OLS model NSE is “poor” since it is close to zero. The MLR model results are “good” and “acceptable” except for four parameter combinations. Therefore, the MLR and OLS model results have some discrepancies. The MLR-R model NSE is different from the OLS model NSE and similar to the MLR model result. The MLR-I model NSE with the SS (no outliers) method has a large range that is closer to one than the NSE values of the other models (Figure 12).

**Figure 13:**
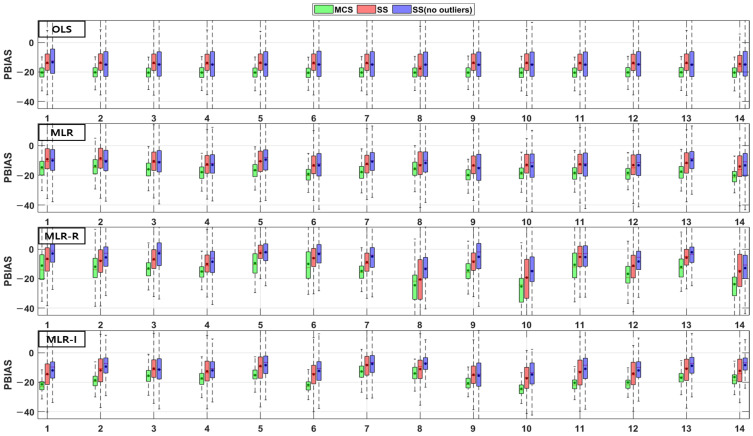
Error analysis (PBIAS) results of TP removal in retention ponds.

**Figure 14:**
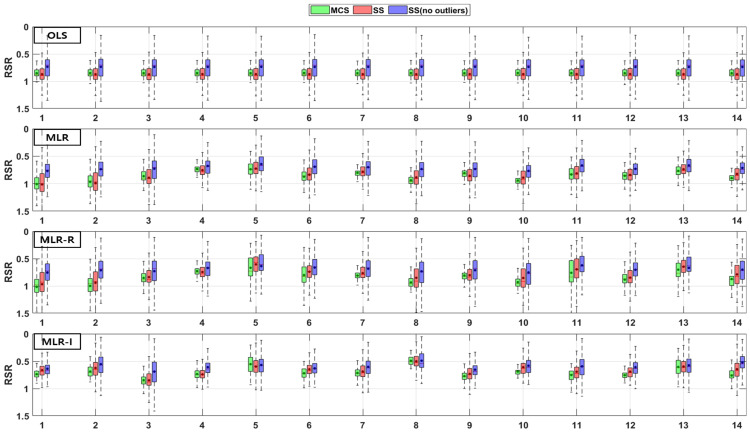
Error analysis (RSR) results of TP removal in retention ponds.

**Figure 15:**
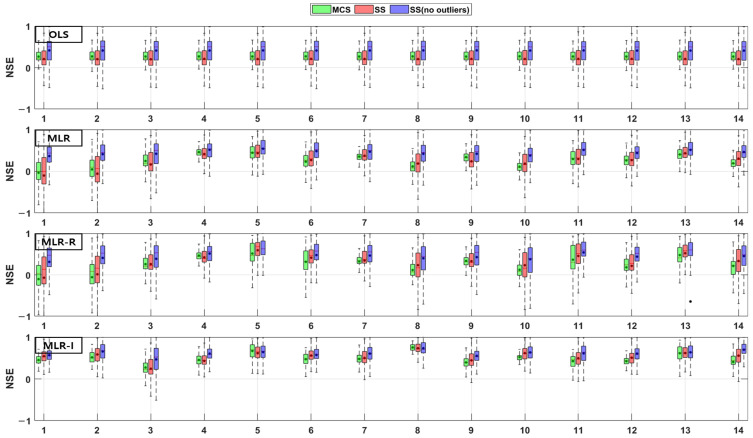
Error analysis (NSE) results of TP removal in retention ponds.

The PBIAS range of values pertaining to the TP removal performance in retention ponds includes some positive values, which indicates that the models exhibit an underestimation bias (Figure 13). The TP removal performance in the retention ponds according to the RSR (Figure 14), which is similar to that in the detention basins (Figure 8), is worse for some parameter combinations in the MLR-R model than in the OLS model. The MLR and MLR-R model RSR values are “poor” and “acceptable” (except with the SS (no outliers) method) since they have negative values (Figure 15). Therefore, the MLR and MLR-R model results (except with the SS (no outliers) method) are worse than the OLS model results, as shown in Figure 15. Moreover, the MLR-I model ranges span all three performance levels.

In comparison to the PBIAS values of the other models, the MLR-R model PBIAS is close to zero in both the detention basins and the retention ponds. Among the MLR models, the MLR-I models RSR and NSE perform better. Some of the MLR-R model RSR and NSE values exhibit worse performance than those of the OLS model among the 14 combinations of parameters. The MLR-I model performs well in the three model evaluations compared with the OLS model.

### 3.2. Combinations of Parameters

**Figure 16:**
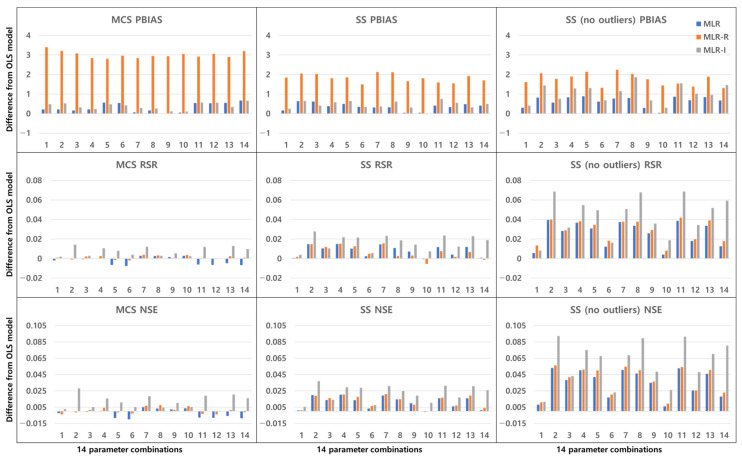
Differences between the OLS model and the other three MLR models by averaging the values of the model evaluations with the three sampling methods for TSS removal in detention basins.

**Figure 17:**
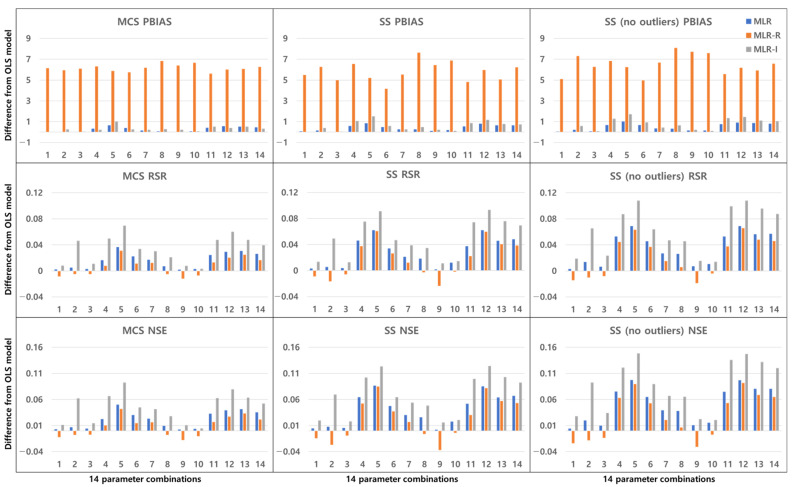
Differences between the OLS model and the other three MLR models by averaging the values of the model evaluations with the three sampling methods for TP removal in detention basins.

**Figure 18:**
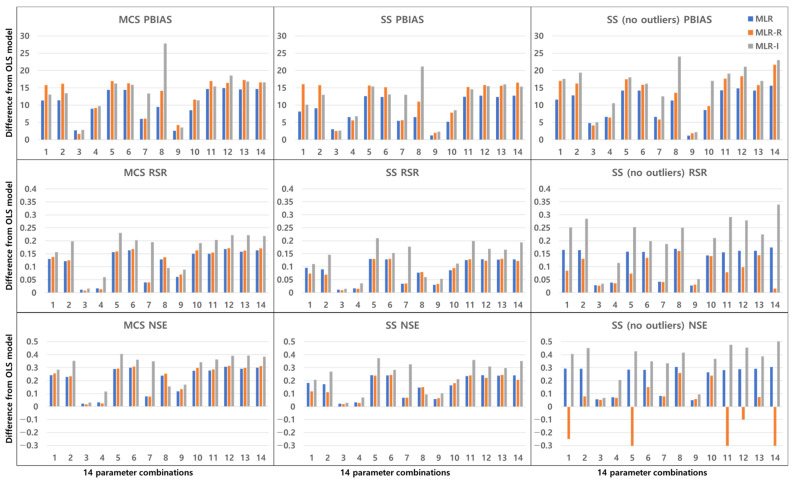
Differences between the OLS model and the other three MLR models by averaging the values of the model evaluations with the three sampling methods for TSS removal in retention ponds.

**Figure 19:**
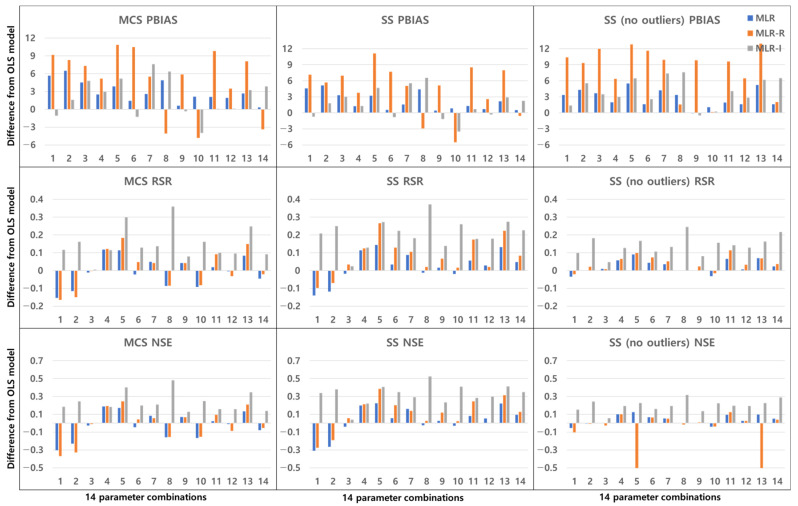
Differences between the OLS model and the other three MLR models by averaging the values of the model evaluations with the three sampling methods for TP removal in retention ponds.

Figure 16, Figure 17, Figure 18 and Figure 19 show the differences between the OLS model and the other three MLR models by averaging the values of the model evaluations with the three sampling methods. Figure 16 shows the TSS removal performance values in the detention basins. The RSR and NSE (but not the PBIAS) have the same trend, although the resulting values are different among the sampling methods. In the PBIAS results, the MLR-R model differs greatly from the OLS model across all sampling methods. Except for some combinations, the RSR and NSE results of the MLR-I model differ strongly from those of the OLS model. Large discrepancies between the RSR and NSE results are found for parameter combinations No. 2, No. 8, and No. 11. Combinations No. 2 and No. 8 contain the flow rate, and combinations No. 2, No. 8 and No. 11 all include the bottom area.

As shown in Figure 17, the TP removal performance in the detention basins in the MLR-R model differs more from that in the OLS model than from any of the other models in the PBIAS results, and the MLR-I model differs from the OLS model in terms of the RSR and NSE. Unlike Figure 16, the PBIAS differs more from the results of the OLS model with the SS (no outliers) method than from the results with the MCS method. In terms of the RSR and NSE, the MLR-I model exhibits the greatest difference among all the sampling methods, and the MLR-R model performs worse than the OLS model for some combinations; i.e., combinations No. 5 and No. 12 show considerable differences from the OLS model, and combinations No. 11 through No. 14 (which include the detention time) have clear differences from the OLS model.

Unlike the PBIAS of the TSS removal performance in detention basins (Figure 16), there is a larger discrepancy among the PBIAS values of the MLR-R and OLS models in the TSS removal performance in retention ponds (Figure 18) for all parameter combinations. However, the MLR-R model is not the best model in the model evaluations in the PBIAS of TSS removal performance in retention ponds (Figure 18). In combinations No. 4, No. 7, and No. 8, the MLR-I model shows a larger PBIAS difference from the OLS model than the MLR-R model with all sampling methods. Except for the SS (no outliers) method, in terms of the RSR and NSE, the MLR-R model in combination No. 8 exhibits better model performance evaluations than the other models. With the SS (no outliers) method, in terms of the NSE, the MLR-R model displays negative values for some combinations. With the SS (no outliers) method, the MLR-I model provides the largest difference from the OLS model in all model evaluations for combination No. 14, whereas with the other sampling methods, the MLR-I model shows discrepancies with the OLS model for combinations No. 5 and No. 11; similar to combination No. 14, combinations No. 3 and No. 9 show small differences from the OLS model in all sampling methods and model evaluations. With all sampling methods in the MLR-I model, combination No. 4 (including the depth and flow rate) has the smallest gap with respect to the OLS model among the combinations that include the flow rate with GI parameters.

In the case of combination No. 8 regarding the TP removal performance in retention ponds (Figure 19), the MLR-I model results are different from the OLS model results with all sampling methods and in all model evaluations. Except for PBIAS, combination No. 8 reveals a large gap between the OLS model and the MLR-I model among all parameter combinations. In terms of the PBIAS, the MLR-R model results are largely different from those of the OLS model for combinations No. 5 and No. 13. However, with the SS (no outliers) method, in terms of the NSE, the MLR-R model shows negative values for combinations No. 5 and No. 13. The SS (no outliers) method features the smallest differences from the OLS model results compared to the other sampling methods, but few models display negative values.

## 4. Discussion

The difference between a detention basin and a retention pond is the existence of a permanent pool, i.e., the former does not have a permanent pool, whereas the latter (being wet ponds) does have a permanent pool. The performance results in retention ponds are more different from the OLS results than those in detention basins. Certain types of BMPs, such as wet ponds, can reduce runoff through evaporation and infiltration. The removal of pollutants contributes to reducing pollutant loading while improving effluent quality [31]. Figure 16 and Figure 18 show that the HLR of retention ponds is correlated with TSS removal. The wetland treatment performance is determined by the inflow, HLR, and detention time. Carleton et al. [12] showed that wetlands had no significant HLR results for TP removal but showed significant results with respect to the detention time and the ratio of watershed area to wetland area. Combination No. 1 in Figure 19 (including the HLR) indicates that the difference from the OLS model is not appreciable compared to other combinations in detention basins. Combination No. 14 (including the detention time and the ratio of the watershed area to the permanent pool area of retention ponds) is different from the OLS model among the 14 combinations, so there is a correlation. These two parameters affect the effluent concentration because combination No. 14 shows a satisfactory difference among all TSS and TP removal results in detention basins and retention ponds.

As shown in Figure 18, when retention pond depth is included in the parameter combinations, the OLS results are similar, unlike the other geometric parameters of detention basins. As the depth increases, the pollution control performance deteriorates because the particles take longer to settle through the larger vertical water column [14,15]. The depth and detention time have an impact on settling by gravity. As shown in Figure 17, in the TP removal results in detention basins, the depth performance is better than that of combinations with the bottom area regardless of the hydraulic parameters, such as the inflow rate and detention time. Walker [32] showed that the removal of phosphorus from urban runoff in detention basins is more sensitive to the pond depth than to the surface area. The results of this study demonstrate that, in addition to the surface area, the bottom area also shows the same trend. 

Combinations No. 2 and No. 11 in Figure 16 and Figure 18 include the area as the GI geometric parameter, and the differences from the OLS model are the largest among the 14 combinations. Park et al. [16] found that the confidence intervals for runoff concentrations decreased with the surface area of detention basins and retention ponds. This means that the TSS effluent concentration decreased as the surface area increased in both the detention basins and the retention ponds. Barrett [4,6] showed that only the influent concentration (and not the permanent pool volume or surface area of retention ponds) was correlated with the mean TSS concentration. However, in Figure 18, combination No. 5 shows a correlation when both the inflow rate and volume are included. In the case of combination No. 9 (including the surface area and influent concentration only), there is no significant difference, but the results confirm that there is a difference from the OLS model in combination with hydrological factors, such as the flow rate and detention time. These differences from the OLS model indicate that other parameters are correlated with the effluent concentration. Therefore, even a small difference indicates some correlation. The model evaluation results obtained with the three sampling methods and the four models in Figure 4, Figure 5, Figure 6, Figure 7, Figure 8, Figure 9, Figure 10, Figure 11, Figure 12, Figure 13, Figure 14 and Figure 15 show that, in addition to the influent concentration, various other parameters are also related to the effluent concentration. Existing studies have concluded that parameters other than the influent concentration do not significantly affect the effluent concentration, but that such effects appear to manifest through differences from the OLS model using only the influent concentration and MLR models that include other parameters. As shown in Figure 16, Figure 17, Figure 18 and Figure 19, the difference from the OLS model is larger in the combinations with GI geometric parameters and the HLR than in the combinations with one parameter in addition to the influent concentration. These figures and results suggest that designing the GI model with consideration of both GI geometric and hydrological parameters can produce better performance than designing the model using only one parameter, such as the influent concentration.

## 5. Conclusions

Three sampling methods, namely, MCS, SS and SS (no outliers), were used to reduce the bias of stormwater events per GI through the removal of TSS and TP from two GIs, i.e., detention basins and retention ponds. This study applied the OLS model (including only the influent concentration) and the MLR, MLR-I and MLR-R models, which included other parameters. Although the detention basins exhibited better model performance evaluations than the retention ponds, the correlations of the parameters showed clearer results in the retention ponds. For both detention basins and retention ponds, the hydrological factors of inflow rate and detention time were positively correlated with GI geometric factors. Combinations with both hydrologic and GI geometric factors showed more of a gap with OLS results than those including only one factor. Among several the GI geometric parameters, area and volume included in the combination were positively correlated with the two GIs. The two GIs were also positively correlated with the watershed area. In TSS removal of detention basins, combinations of the bottom area included hydrological factors and the ratio of the bottom area to the watershed area had more difference than other combinations. Therefore, the bottom area was more relevant than other GI geometric parameters in TSS removal of detention basins. In TSS and TP removal of retention ponds, combinations which included depth had smaller gap with OLS results than other combinations with GI geometric factors. But in TSS and TP removal of detention basins, combinations with depth produced different OLS results, similarly to other combinations with GI geometric factors. Unlike detention basins, there was a negative correlation with depth in the retention ponds. This study demonstrates that parameters other than the influent and effluent concentrations are correlated with the treatment performance of detention basins and retention ponds. The combinations with both GI geometric and hydrological parameters showed greater differences from the OLS model than the combinations with only one parameter in addition to the influent concentration. To design the optimal GI model, this study proposed that it would be better to consider other parameters in addition to the influent concentration. For future study, it is necessary to account for the underlying soil condition in the analysis. The type of underlying soil is one of the most important factors involved in determining the performance of a stormwater basin. A basin with a soil with high permeability performs better than one with a less permeable soil. Mechanisms such as adhesion and sedimentation were not considered in combination with the soil type when analyzing the data.

## Figures and Tables

**Table 1:** Stratified sampling values for each parameter.

GI		Area (m^2^)	Depth (m)	Volume (m^3^)	Watershed Area (km^2^)	FlowRate (m^3^/s)	Detention Time (s)	HLR (m/s)	Influent Concentration (C_in_) (mg/L)
Detention Basins	TSS	800	0.4	200	20,000	0.001	20,000	2.0 × 10^−6^	30
	TP	250	0.7	600	40,000	0.004	30,000	5.0 × 10^−6^	0.1
Retention Ponds	TSS	1000	0.35	1000	100,000	0.01	20,000	1.0 × 10^−6^	50
	TP	4000	0.4	2000	300,000	0.05	50,000	1.0 × 10^−6^	0.5

**Table 2:** Model evaluation criteria thresholds [17].

Criteria	PBIAS	RSR	NSE
Good	−10% < PBIAS < 10%	RSR < 0.80	NSE > 0.6
Acceptable	−25% < PBIAS < 25%	0.80 < RSR < 0.98	0.4 < NSE < 0.6
Poor	PBIAS < −25%, PBIAS > 25%	RSR > 0.98	NSE < 0.4

**Table 3:** Combination numbers.

Numbers	1	2	3	4	5	6	7	8	9	10	11	12	13	14
Combinations	C_in_, HLR	C_in_, Q, A	C_in_, Q	C_in_, Q, D	C_in_, Q, V	C_in_, T	C_in_, Q, WA	C_in_, Q, A/WA	C_in_, A	C_in_, A/WA	C_in_, T, A	C_in_, T, D	C_in_, T, WA	C_in_, T, A/WA
